# PCR detection and analyzis of potentially zoonotic Hepatitis E virus in French rats

**DOI:** 10.1186/1743-422X-11-90

**Published:** 2014-05-15

**Authors:** Frederik Widén, Florence Ayral, Marc Artois, Ann-Sophie Olofson, Jay Lin

**Affiliations:** 1 Department of Virology, Immunobiology and Parasitology, The National Veterinary Institute (SVA), 751 89 Uppsala, Sweden; 2 VetAgro Sup – Campus Vétérinaire de Lyon, 1 avenue Bourgelat, 69280 Marcy L’Etoile, France; 3 Department of Biomedical Science and Veterinary Public Health, The Swedish University of Agriculture, 750 07 Uppsala, Sweden

**Keywords:** Hepatitis E, Rat, Zoonoses, PCR

## Abstract

**Background:**

Hepatitis E virus has been detected in a wide range of animals. While Genotypes 1-2 of this virus infect only humans, 3-4 can spread from animals to humans and cause sporadic cases of human disease. Pig, and possibly also rats, may act as a reservoir for virus. From a public health perspective it is important to clarify the role of rats for infection of humans. Rats often live close to humans and are therefore of special interest to public health. Rats live of waste and inside the sewage system and may become infected. Reports of hepatitis E virus in rats have been published but not from France. The possibility that rats in an urban area in France were Hepatitis E virus infected, with which type and relationship to other strains was investigated. This study provides information important to public health and better understanding the occurrence of hepatitis E virus in the environment.

Eighty one rats (*Rattus Norvegicus*) were captured, euthanized, sampled (liver and faeces) and analyzed by real-time RT-PCR’s, one specific for Hepatitis E virus in rats and one specific for genotype 1-4 that that is known to infect humans. Positive samples were analyzed by a nested broad spectrum RT-PCR, sequenced and compared with sequences in Genbank.

**Findings:**

Twelve liver and 11 faeces samples out of 81 liver and 81 faeces samples from 81 captured rats were positive in the PCR specific for Hepatitis E virus in rats and none in the PCR specific for genotype 1-4. Comparison by nucleotide BLAST showed a maximum of 87% similarity to Hepatitis E virus previously detected in rats and significantly less to genotype 1-4.

**Conclusions:**

This is the first study demonstrating that rats in France carries hepatitis E virus and provide information regarding its relation to other virus strains previously detected in rats and other host animals world-wide. Genotype 1-4 was not detected.

## Background

It has been estimated that wildlife is responsible for 72% of emergent infectious diseases in humans [1,2]. In order to limit the spill over of zoonotic agents, improved wildlife pathogen surveillance is required. It is also necessary to gain better insight into viral factors for species specificity. This is true also for Hepatitis E virus (HEV) a pathogen that is present in wildlife, domestic animals and humans [3,4]. In developing countries, it causes large scale disease outbreaks in humans as well as endemic infections related to poor sanitary conditions. In countries with good sanitary standards the disease in humans, Hepatitis E, occurs sporadically. Such infections are either acquired during travelling to endemic areas or from a domestic source in the infected person’s home country [3,4]. Wild-boars or pigs may constitute such a reservoir [3,4] but other species can also play a role. HEV infecting humans only or humans and other mammals are taxonomically divided in four genotypes [5]. While the large scale epidemics and endemic infections in third world countries as well as cases imported from these countries are caused by hepatitis E of genotype 1 or 2, infecting humans only, the domestically acquired infections in industrialized countries are caused by genotype 3 in Europe and North America or 4 in East Asia. It is widely believed that pigs and wild boars constitute a reservoir for genotype 3 or 4 for human infections and humans may acquire these infections through consumption of undercooked pig, wild boar or deer meat. In France the prevalence of anti-HEV antibodies in the general population was determined to be 3.2%, a figure similar to other industrializes countries [6]. A survey performed on French pig farms demonstrated a 65% prevalence of anti-HEV antibodies [7]. Another survey on wild boar demonstrated a prevalence of anti-HEV antibodies between 7.2 and 22.7% in different geographical regions [8]. No figures for Lyon were available but the highest value came from Aveyron that is relatively close to Lyon. The transmission of HEV between different animal species has not yet been clarified. Furthermore it is not known what determines the pathogenicity and the host range. The current taxonomy is based on a study by Lu et al. [5] but has been challenged and additional genotypes have been proposed [9]. HEV has been detected in several species like for example pig, wild boar, deer, moose, rabbit, ferret, mink, rat, poultry and cutthroat trout [3,4,10-15]. HEV from pig and wild boar belong to genotype 3 or 4 and are related. HEV from deer belong to genotype 3 [4] and HEV from rabbits is closely related to genotype 3 [9] while HEV from moose does not belong to genotype 1-4 [10]. Avian HEV share only 50% nucleotide identity with genotype 1-4 and cutthroat trout HEV is very distantly related to the others [14,15]. Rats can be infected with seemingly rat specific HEV or with genotype 3 [13,16]. The zoonotic potential of rat specific HEV is controversial [17,18]. Serological investigations have suggested that humans may be infected by rat specific HEV [19]. It is likely that genotype 1-4 infecting rats can also infect humans. Because *R. norvegicus* is a synanthropic species, humans and rats live in close proximity. Rats are well known to transmit pathogens to humans and other animals. Their high prevalence and propensity to carry pathogens make them a potential reservoir for human pathogens including HEV. Indeed, according to previous surveys, antibodies against HEV are highly prevalent in rats [17] and HEV RNA has been detected in rats (*R. norvegicus* and other rat species) in Germany, USA, Vietnam, Denmark, China and Indonesia [13,16,20-23]. Differentiation of rat specific HEV from HEV genotype 3 by serology has been published [19] but is not generally available. Most HEV strains found in rats were of the rat specific type but genotype 3 RNA has also been detected in rats [16]. It is therefore important to further clarify the role rats may have as a reservoir for human HEV infections. The objectives of this study were to investigate if HEV could be detected in French rats in an urban environment, to determine the type HEV that was present in order to better judge the risk for zoonotic infections and finally to determine the relationship to other previously detected HEV strains order to create a more complete picture of HEV infecting rats.

The study was part of a survey of infectious agents in wild rats (*Rattus Norvegicus*) conducted in the city of Lyon in Center-East France as part of the EU-funded Wildtech project. As a part of this survey, liver and faeces samples from rats were collected and analyzed for presence and characterization of HEV strains.

## Results and discussion

### TaqMan^®^ assay specific for HEV from rat

Eighty-one rats of the species *Rattus Norvegicus* were caught. No other rat species were observed. Twelve of eighty-one (15%) liver samples were positive in real-time RT-PCR amplifying HEV from rat [17]. The rats were labeled “Rat HEV Ly Id number 2012” and the rats with PCR positive liver samples had Id numbers 839, 848, 867, 873, 874, 877, 879, 880, 882, 883, 888, 894. Testing of eighty-one faeces samples resulted in eleven positive samples. The positive faeces samples came from rats that also had positive liver samples. All positive samples came from rats caught in the low income area. Nine (75%) adult male and 3 (25%) adult female rats were positive in this PCR. No positive juvenile rats were found.

The sex ratio of the positive rats can be explained by the fact that 74% of the rats collected in this area were males. Similarly the age ratio of the positive rats (100% adults) can be explained by the clear dominance of adults (88%) among the collected rats.

### Prevalence and confidence interval of HEV specific for rats

Fifteen percent (p = 12/81) of the rat tested for HEV had a PCR-positive result. Then the 95% confidence interval for the prevalence was *IC*_95%_ = [7%–22%].

### TaqMan^®^ assay specific for genotype 1-4 of HEV

All liver and faecal samples tested by a real-time PCR specific for genotype 1-4 [24] were found to be negative in this assay.

### Nested PCR of real-time RT-PCR positive samples

All samples positive by real-time RT-PCR for HEV from rat were positive when amplified with this nested PCR [25].

#### Sequence analysis

Assembly of sequences (DNASTAR Lasergene 8) and subsequent BLAST analysis confirmed that the sequences represented HEV and were similar to other HEV sequences recovered from rats.

**Figure 1:**
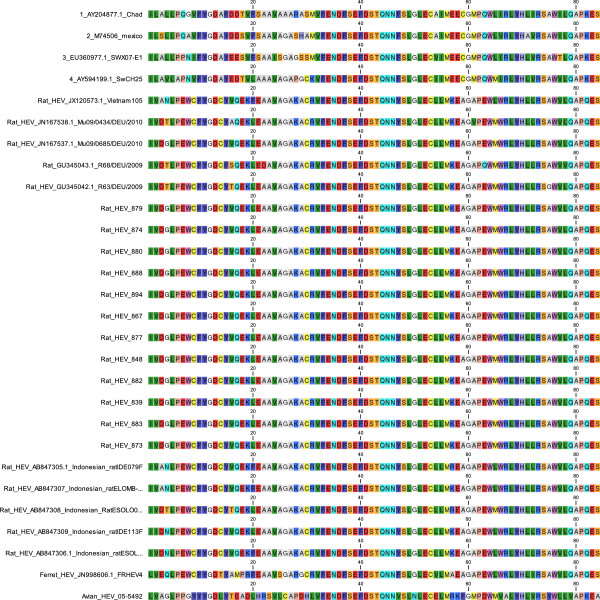
**Multiple sequence alignment of deduced amino acid sequences from nucleotide sequences used for phylogeny (Figure**2**).** The multiple alignment was constructed using MEGA5. It corresponds to the same fragment used for phylogeny (Figure 2).

The sequences from the twelve rats displayed highly similar sequences. Only one nucleotide position displayed synonymous substitution. The HEV strain from rats 874, 883 and 894 had a “C” at position 4192, as compared to the HEV sequence from rat, rat/Mu/0685/DEU2010, accession number JN167537.1. Rat 877 had a wobble base that varied between “C” and “T” (R) at this position while the other sequences had a “T” at position 4192 in the cDNA sequence. The multiple sequences deduced from each individual rat sample confirmed this single silent mutation. The high level of similarity between these sequences can be explained by the fact that all HEV positive rats were captured at the same location. Nucleic acid BLAST analyses against HEV sequences at NCBI gave a maximum identity of 87%, for the entire (100%) amplicon sequence, as compared to the German HEV strain rat/Mu09/0685/DEU/2010 followed by identities between 80% and 86% for other strains from German, American, Chinese and Vietnamese rats. The strain detected in Vietnam (HEV strain Vietnam-105, accession number JX120573.1), had 82% identity. Two strains from USA displayed an identity of 84% (HEV rat/USA/2003, accession number JF516246.1) and 83% (Hepatitis E virus isolate MVZ201020, accession number JQ898482.1). Several Chinese strains displayed identities of 83%. Although the difference to other strains from rat, by nucleotide sequence BLAST was 12%-20%, the difference by protein BLAST was very small (Maximum 98% identity, HEV strain KS12/1305, Denmark, accession number AGH06684). The high similarity between rat HEV strains is also evident in the amino acid alignment (Figure 1). All strains from rats collected in this study are 100% identical in the amino acid alignment and they differ by a few amino acids only when compared to other rat HEV strains. However when compared to genotype 1-4 the difference is significantly larger and largest when compared with avian HEV.

**Figure 2:**
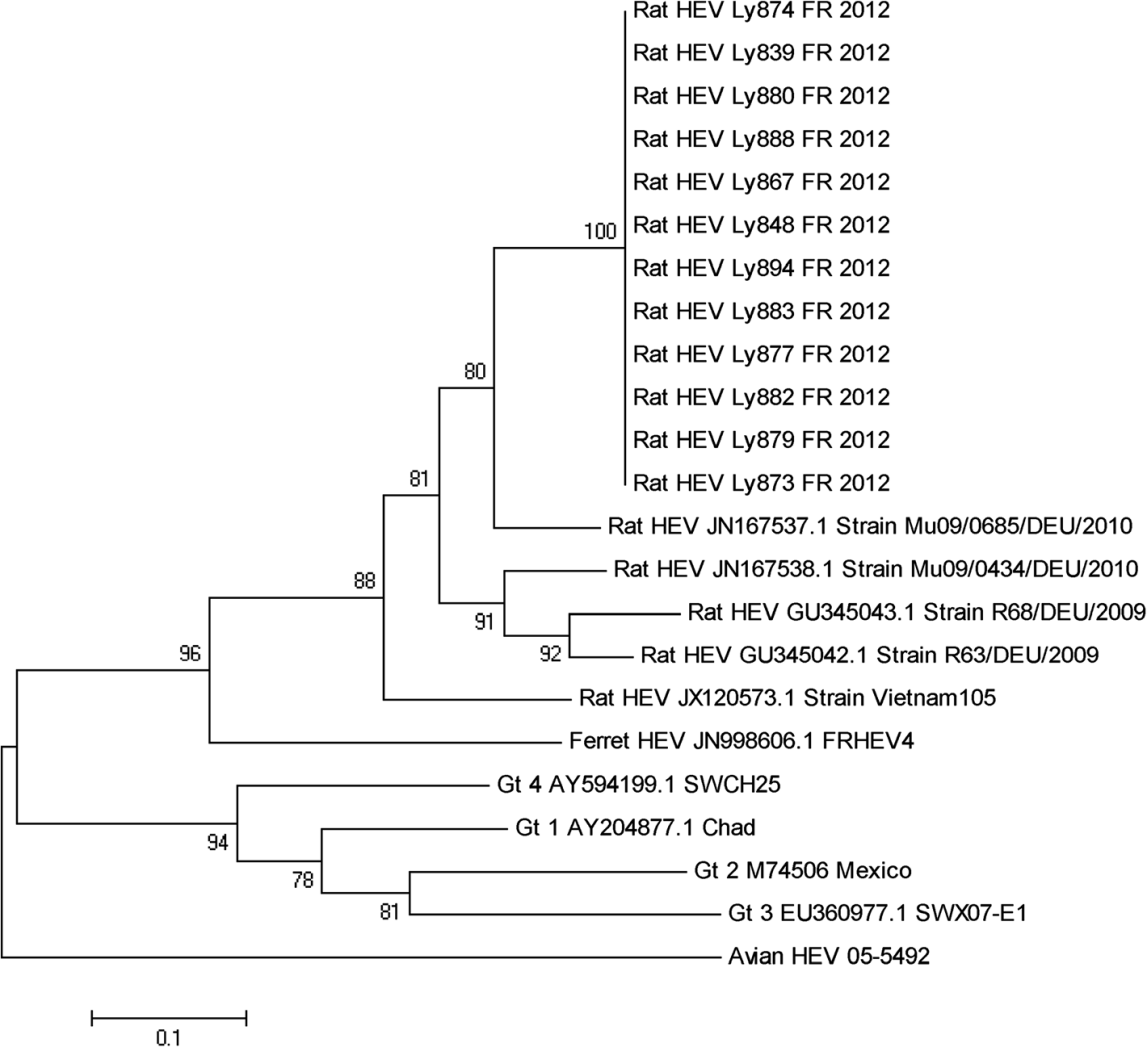
**Phylogenetic tree depicting the relation between the HEV sequences from French rats to a selection of other HEV sequences.** Phylogenetic tree of sequences corresponding to a 254 nt long fragment from the nested PCR product of the amplified RdRp fragment. The sequence corresponds to nucleotide position 4127 to 4371 of the rat HEV strain rat/Mu/0685/DEU2010, accession number JN167537.1. The tree was constructed by the Neighbor joining method using MEGA 5.05. The tree is depicting the relationship of the French HEV strains from 12 rats, here called “Rat HEV Ly, sample number, and Fr 2012” and described in this article, with selected HEV sequences from rat, ferret, avian HEV and genotype 1-4. The bar indicates the evolutionary distance as number of base substitutions per site. The bootstrap consensus was generated using 1000 replicates. Branches corresponding to partitions reproduced in less than 50% bootstrap replicates were collapsed. The percentage of replicate trees in which the associated taxa clustered together in the bootstrap test is shown. The tree is drawn to scale. The evolutionary distances were computed using the Tamura-Nei method (number of base substitutions per site). The rate variation among sites was modeled with a gamma distribution (shape parameter = 6). All positions containing gaps and missing data were eliminated.

The phylogenetic tree (Figure 2) confirmed that the HEV strains detected in this study are most related to other Rat specific HEV strains but cluster separately from other rat HEV strains. They are most related to Rat HEV strains detected in Germany (Figure 2) while the strain detected in Vietnam is more distant.

HEV of genotype 3 has previously been detected in rats [14]. These samples were also tested for genotype 1-4 with an assay that is regarded as highly sensitive. Because the rats were negative for genotype 3 and positive for rat related HEV only, they do not pose an evident risk for zoonotic HEV infections of humans. The HEV detected in these rats is related to other HEV strains from rats. The difference compared to HEV genotype 3 is considerably larger at the protein level (maximum 74% identity) while the similarity is higher when compared with HEV from ferrets (maximum 86% identity). The phylogenetic comparison (Figure 2) confirms that detected HEV sequences clusters together with other strains from rats and is not so distant from the HEV strain from ferret [10]. However, the detected strains do not cluster closely with genotype 1-4. On the contrary, genotype 1-4 is clustering on a branch that is separated from the branch containing rat and ferret HEV strains. While it has been clearly demonstrated that genotype 1-4 certainly poses a risk for human infections there is also a group of mammalian HEV strains that do not belong to genotype 1-4. The number of strains in this group has been expanding rapidly as an increasing number of species have been screened for HEV. While some strains like Avian HEV and HEV in cutthroat are only distantly related to HEV infecting mammals, there are a number of HEV strains that are more similar to genotype 1-4 but still sufficiently different not to be included in any of these genotypes, like for example rat and ferret HEV. These strains are currently not regarded as zoonotic. However, further studies of determinants of strain specificity, pathogenicity and the relationship between different mammalian HEV strains may shed more light on light on the zoonotic capability of HEV.

## Methods

### Collection of samples from rats (Rattus Norvegicus)

Rats provided for this study were trapped for the purpose of pest control (trapping agreement n°691810). They were captured, euthanized and sampled following the relevant ethical and safety rules (animal research agreement n° 69-020931). The procedure was supervised by the ethical committee of the VetAgro Sup and European regulation (EU Directive 86/609).

The necessary sampling size (n ≈ 90) was calculated using 95% confidence level, a relative accuracy of 50% and 10%-30% expected prevalence.

The survey was conducted in selected areas of Lyon during a six month period from October 2011 to March 2012. The trapping sites were chosen for their abundance of rats, as reported by the Hygiene Service of the city and their environmental differences relevant for the risk investigations. A peri-urban area, a low income area, a public garden, a waste treatment plant (WTP) and a waste water treatment plant (WWTP), were the five trapping areas.

The low income area comprised of 619 apartments which were distributed on 0.6 km^2^ and the traps were set in 20% (n = 127) of the dwellings. The public garden was situated in the city centre and featured a large pond and captive wildlife. In the industrial area, the trapping was performed in a WTP and a WWTP. The trapping success was 42, 8, 23, 7 and 1 captured Norway rats in the low income area, the public garden, the WTP, the peri-urban area and the WWTP plant, respectively. A total of 81 (56 male and 25 female) rats were captured and screened by HEV PCR. Of these 54 were adults (as defined by a weight over 100 gram and having sexually mature organs) and 27 juvenile. The captured rats were labelled “Rat HEV Ly Id number 2012”.

### Trapping and sampling of rats

All rats were captured in small (28 cm × 9 cm × 9 cm) or large (50 cm × 15 cm × 15 cm) single catch rat traps. The traps were placed on the paths of rats as described by people familiar with the area (visitors, employees, inhabitants). The distance between each trap depended on the area to be covered and varied from 1 to 5 m. Traps were baited with peanut butter and set at locations around the clock for the trapping period. The rats were collected each morning. Captured rats were transported in the traps to the laboratory, placed inside fume hoods and immediately anesthetized using isofluran and euthanized by cervical dislocation. The species, weight, sex and approximate age (juvenile vs adults) of each rat was determined by ocular inspection of morphology and weighing. Eighty-one rats were aseptically dissected and two liver samples of 30 mg each and two faeces samples were collected (from the rectum). The samples were immediately stored at -80°C prior to shipping to SVA in Sweden.

### Extraction of RNA

Eigthy-one livers samples and 81 faeces samples from 81 rats were analyzed. The liver samples were cut and the fresh surfaces were sampled using a cotton swab which was subsequently soaked in 850 μl TE-buffer. The faeces samples were diluted 1:4 in TE-buffer. RNA was extracted from 90 μl liver sample suspension using a magnatrix (Magnetic Biosolutions Sweden) or from 50 μl faeces samples suspension using a MagMax (King Fisher) Robot.

### Reverse transcription of RNA and PCR amplification of HEV RNA in rat samples

Eighty-one liver and faeces samples (on from each from each rat) were tested by PCR as specified below.

#### TaqMan^®^ assay specific for HEV from rat

Extracted RNA, including positive control R68, was analyzed by Taq Man real-time RT-PCR specific for HEV from rat, on a Corbett Rotorgene 3000 instrument, as previously described [17] but modified according to the recommended cycling parameters given in the instructions for the Agpath rRT PCR kit (Life technologies, USA) and with modified reverse primer (rHEV-R2) and probe (rHEV-P2) sequence as recommended by Dr. R. Johne (personal communication) to avoid false negative results. The PCR target the region 5214-5286 in the rat/Mu/0685/DEU2010 sequence. Briefly; the forward primer rHEV-F (5′-TACCCGATGCCGGGCAGT-3′), the reverse primer rHEV-R2 (5′-ATCYACATCWGGGACAGG-3′) and the probe rHEV-P2 (5′-AATGACAGCACAGGCACCGGCGCC-3′) labelled with 6-FAM at the 5′ end and Black hole quencher (BHQ) at the 3′ end was used. The total reaction volume was 25 μl per tube including 4.6 μl of added template. After reverse transcription for 10 min. at 50°C and inactivation for 15 min. at 95°C, 55 cycles consisting of 15 sec. at 95°C and 60 sec. at 60°C was performed. Fluorescence was collected during the annealing step.

#### TaqMan^®^ assay specific for genotype 1-4 of HEV

Subsequently, all liver and faecal samples, including positive control Swe 8, were analyzed by a PCR assays specific for genotype 1-4 as previously described [24] with modifications (see below). The PCR target approximately position 5060 to 5126 in the rat/Mu/0685/DEU2010 and position 5293 to 5362 in the SwX07-E1 sequence. Briefly, the forward primer JHEVF (5′-GGTGGTTTCTGGGGTGAC-3′), the reverse primer JHEVR (5′-AGGGGTTGGTTGGATGAA-3′) and the probe JHEVP (5′-CCGACAGAATTGATTTCGTCGGC-3′) labelled with Cy5 at the 5′ end and Black hole quencher 2 (BHQ2) at the 3′ end was used. The total reaction volume was 12.5 μl per tube including 3 μl added template. The Agpath rRT PCR kit was used for the RT-PCR and the RT-PCR program was identical with the RT-PCR program specific for HEV from rat.

#### Nested PCR of real-time RT-PCR positive samples

The twelve samples positive by real-time RT-PCR specific for rat HEV were amplified with a previously described nested PCR targeting a fragment of the RNA dependent RNA polymerase (RdRp) in ORF1 [25]. Briefly, extracted RNA was used as template for a one-step RT-PCR (Qiagen, Germany). Primers HEV-cs (5′-TCGCGCATCACMTTYTTCCARAA-3′) and HEV-cas (5′-GCCATGTTCCAGACDGTRTTCCA-3′) were used for the RT-PCR while the modified primer HEV-csn mod (5′-TGTTGCCCTGTTTGGCCCCTGGTTTAG-3′) and the primer HEV-casn (5′-CCAGGCTCACCRGARTGYTTCTTCCA-3′) were used for the nested step. For the nested PCR, the Platinum Taq (Life Technologies) was used. For the one-step RT-PCR the reactions were held at 50°C for 30 min., and at 95°C for 15 min. followed by 40 cycles of 94°C for 30 sec., 50°C for 30 sec. and 72°C for 45 sec. followed by final extension at 72°C for 10 min.. For the nested PCR the reactions were activated at 94°C for 30 sec. followed by 35 cycles of 94°C for 30 sec., 50°C for 30 sec. and 72°C for 45 sec. followed by final extension at 72°C for 10 min.

### Estimation of prevalence

The estimated prevalence ($\hat{p}$) in the rat population within the sampled area here called “the low income area”, in Lyon, was the proportion of positive samples. The approximate 95% confidence interval (IC_95%_) of the true prevalence was given by ${\mathrm{IC}}_{95\%}=p\pm 1.96*\sqrt{\frac{\mathrm{pq}}{n}}$ with “p” the proportion of PCR-positive rats, “q” the proportion of PCR-negative rats and “n” the sample size.

### Sequencing of HEV positive samples

The 12 samples successfully amplified by nested PCR were sequenced from different PCR products on average 4-5 times per product using the inner primers from the nested PCR. The sequencing was performed at an Applied Biosystem 3130×l.

### Sequence analysis

Multiple alignments of sequences were performed using DNA-STAR Lasergene 8 and a 254 nt fragment was selected for further analysis. This fragment was chosen because it was the largest that had good sequence coverage for all 12 rat samples. Consensus sequences for individual rat samples were created with DNASTAR Lasergene 8. These consensus sequences were analyzed using the nucleotide BLAST program at NCBI as well as by Mega 5.05.

Phylogenetic and molecular evolutionary analyses were conducted using *MEGA* version 5.05. The phylogenetic tree was deduced using Neighbour joining with Tamura 3 parameter and gamma parameter 0.3. Bootstrap analysis was performed with 1000 repeats.

## Ethical statement

Information regarding approval by an ethics committee is given in the manuscript. Rats provided were trapped for the purpose of pest control (agreement n°691810) and captured, euthanized and sampled following the ethical rules (agreement n° 69-020931) supervised by the ethical committee of the VetAgro Sup and European regulation (EU Directive 86/609).

## Competing interests

The authors declared that they have no competing interests.

## Authors’ contributions

FW planned and carried out the molecular genetic studies and drafted the manuscript. FA planned the study, took part in sample collection, provided the samples for molecular analysis and took part in drafting the manuscript. MA conceived and planned the study and corrected the manuscript. ASO planned and performed molecular analysis and corrected the manuscript. JL performed sequence alignments, designed and performed phylogenetic analysis and helped with drafting of the manuscript. All authors read and approved the final manuscript.

## References

[B1] Cleaveland S Laurenson MK Taylor LH Diseases of humans and their domestic mammals: pathogen characteristics, host range and the risk of emergence Philos Trans R Soc Lond B Biol Sci 2001 356 991 999 10.1098/rstb.2001.0889 11516377 PMC1088494

[B2] Jones KE Patel NG Levy MA Storeygard A Balk D Gittleman JL Daszak P Global trends in emerging infectious diseases Nature 2008 451 990 993 10.1038/nature06536 18288193 PMC5960580

[B3] Purcell RH Emerson SU Hidden danger: the raw facts about hepatitis E virus J Infect Dis 2010 202 819 821 10.1086/655900 20695795 PMC2941993

[B4] Arends JE Ghisetti V Irving W Dalton HR Izopet J Hoepelman AIM Salmon D Hepatitis E: an emerging infection in high income countries J Clin Virol 2014 59 81 88 10.1016/j.jcv.2013.11.013 24388207

[B5] Lu L Li C Hagedorn CH Phylogenetic analysis of global hepatitis E virus sequences: genetic diversity, subtypes and zoonosis Rev Med Virol 2006 16 5 36 10.1002/rmv.482 16175650

[B6] Boutrouille A Bakkali-Kassimi L Cruciére C Pavio N Prevalence of Anti-Hepatitis E Virus antibodies in French blood donors J Clin Microb 2007 45 2009 2010 10.1128/JCM.00235-07 PMC1933073 17460057

[B7] Rose N Lunazzi A Dorenlor V Merbah T Eono F Eloit M Madec F Pavio N High prevalence of Hepatitis E virus in French domestic pigs Comp Immunol Microbiol Infect Dis 2011 34 419 427 10.1016/j.cimid.2011.07.003 21872929

[B8] Payne A Rossi S Lacour SA Vallée I Garin-Bastuji B Simon G Hervé S Pavio N Richomme C Dunoyer C Bronner A Hars J Bilan sanitaire du sanglier vis-à-vis de la trichinellose, de la maladie d’Aujeszky, de la brucellose, de l’hépatite E et des virus influenza porcins en France Bulletin Epidemiologique Santé Animal – Alimentation 2011 44 2 8

[B9] Smith DB Purdy MA Simmonds P Genetic variability and the classification of hepatitis E virus J Virol 2013 87 4161 4169 10.1128/JVI.02762-12 23388713 PMC3624379

[B10] Lin J Norder H Uhlhorn H Belák S Widén F Novel hepatitis E like virus found in Swedish moose J Gen Virol 2014 95 557 570 10.1099/vir.0.059238-0 24296469 PMC3929172

[B11] Izopet J Dubois M Bertagnoli S Lhomme S Marchandeau S Boucher S Kamar N Abravanel F Guérin JL Hepatitis E virus strains in rabbits and evidence of a closely related strain in humans, France Emerg Infect Dis 2012 18 1274 1281 22840216 10.3201/eid1808.120057 PMC3414036

[B12] Raj VS Smits SL Pas SD Provacia LBV Moorman-Roest H Osterhaus ADME Haagmans BL Novel Hepatitis E Virus in Ferrets, the Netherlands Emerg Infect Dis 2012 18 1369 1370 10.3201/eid1808.111659 22840220 PMC3414025

[B13] Johne R Heckel G Plenge-Bonig A Kindler E Maresch C Reetz J Schielke A Ulrich RG Novel hepatitis E virus genotype in Norway rats, Germany Emerg Infect Dis 2010 16 1452 1455 10.3201/eid1609.100444 20735931 PMC3294985

[B14] Huang FF Sun ZF Emerson SU Purcell RH Shivaprasad HL Pierson FW Toth TE Meng XJ Determination and analysis of the complete genomic sequence of avian hepatitis E virus (avian HEV) and attempts to infect rhesus monkeys with avian HEV J Gen Virol 2004 85 1609 1618 10.1099/vir.0.79841-0 15166445

[B15] Batts W Yun S Hedrick R Winton J A novel member of the family Hepeviridae from cutthroat trout (Oncorhynchus clarkii) Virus Res 2011 158 116 123 10.1016/j.virusres.2011.03.019 21458509

[B16] Lack JB Volk K Van Den Bussche RA Hepatitis E virus genotype 3 in wild rats, United States Emerg Infect Dis 2012 18 1268 1273 22840202 10.3201/eid1808.120070 PMC3414038

[B17] Johne R Dremsek P Kindler E Schielke A Plenge-Bonig A Gregersen H Wessels U Schmidt K Rietschel W Groschup MH Guenther S Heckel G Ulrich RG Rat hepatitis E virus: Geographical clustering within Germany and serological detection in wild Norway rats (Rattus norvegicus) Infect Genet Evol: J Mol Epidemiol Evol Genet Infect Dis 2012 12 947 956 10.1016/j.meegid.2012.02.021 22554648

[B18] Cossaboom CM Córdoba L Sanford BJ Piñeyro P Kenney SP Dryman BA Wang Y Meng XJ Cross-species infection of pigs with a novel rabbit, but not rat, strain of hepatitis E virus isolated in the United States J Gen Virol 2012 93 1687 1695 10.1099/vir.0.041509-0 22535776 PMC3541760

[B19] Dremsek P Wenzel JJ Johne R Ziller M Hofmann J Groschup MH Werdermann S Mohn U Dorn S Motz M Mertens M Jilg W Ulrich RG Seroprevalence study in forestry workers from eastern Germany using novel genotype 3- and rat hepatitis E virus-specific immunoglobulin G ELISAs Med Microbiol Immunol 2012 201 189 200 10.1007/s00430-011-0221-2 22179131

[B20] Li TC Ami Y Suzaki Y Yasuda SP Yoshimatsu K Arikawa J Takeda N Takaji W Characterization of full genome of rat hepatitis E virus strain from Vietnam Emerg Infect Dis 2013 19 115 118 10.3201/eid1901.121007 23260149 PMC3558001

[B21] Wolf S Reetz J Johne R Heiberg AC Petri S Kanig H Ulrich RG The simultaneous occurrence of human norovirus and hepatitis E virus in a Norway rat (Rattus norvegicus) Arch Virol 2013 158 1575 1578 10.1007/s00705-013-1646-2 23443935

[B22] Li W Guan D Su J Takeda N Wakita T Li TC Ke CW High prevalence of rat hepatitis E virus in wild rats in China Vet Microbiol 2013 165 275 280 10.1016/j.vetmic.2013.03.017 23623690

[B23] Mulyanto Suparyatmo JB Andayani IG Khalid Takahashi M Ohnishi H Jirintai S Nagashima S Nishizawa T Okamoto H Marked genomic heterogeneity of rat hepatitis E virus strains in Indonesia demonstrated on a full-length genome analysis Virus Res 2014 179 102 112 24231359 10.1016/j.virusres.2013.10.029

[B24] Jothikumar N Cromeans TL Robertson BH Meng XJ Hill VR A broadly reactive one-step real-time RT-PCR assay for rapid and sensitive detection of hepatitis E virus J Virol Methods 2006 131 65 71 10.1016/j.jviromet.2005.07.004 16125257

[B25] Johne R Plenge-Bönig A Hess M Ulrich RG Reetz J Schielke A Detection of a novel hepatitis E-like virus in faeces of wild rats using a nested broad-spectrum RT-PCR J Gen Virol 2010 91 750 758 10.1099/vir.0.016584-0 19889929

